# Concurrent Validity and Agreement of Viewpoint-Dependent Markerless Motion Tracking in Upper-Limb Assessment: A Validation Against Optoelectronic Motion Capture

**DOI:** 10.3390/s26196248

**Published:** 2026-10-01

**Authors:** Suman Shrestha, Yuichiro Honda, Haruki Toda, Takaaki Chin, Mitsuru Irie

**Affiliations:** 1 Graduate School of Engineering, Osaka Sangyo University, Daito 574-8530, Osaka, Japan; 2 Department of Research and Development, The Hyogo Institute of Assistive Technology, Kobe 651-2181, Hyogo, Japan; haruki-toda@nuhw.ac.jp (H.T.); chin@irie.or.jp (T.C.); 3 Department of Systems Engineering, Faculty of Systems Engineering, Osaka Sangyo University, Daito 574-8530, Osaka, Japan; honda@eic.osaka-sandai.ac.jp

**Keywords:** markerless motion capture, upper limb kinematics, MediaPipe, rehabilitation engineering, concurrent validity

## Abstract

Quantitative upper-limb assessment in rehabilitation traditionally relies on expensive optoelectronic reference tracking. While single-camera markerless systems offer an accessible alternative, their viewpoint-dependent accuracy and depth ambiguity remain critical concerns. This study systematically validates an integrated MediaPipe tracking pipeline (Pose and Hands) against an 8-camera optoelectronic system. Ten healthy males performed four clinical upper-limb tasks, recorded simultaneously across three spatial camera viewpoints (frontal, oblique elevated, and oblique side). To isolate spatial accuracy, validations utilized direct unmodeled geometric tracking vectors rather than clinical joint models. Results demonstrate that agreement and accuracy are systematically dependent on movement geometry and camera perspective. Planar tasks like shoulder abduction exhibited moderate to good peak range-of-motion agreement (*ICC* = 0.63 − 0.82) and precision (RMSE = 9.00^∘^ − 11.23^∘^). For transverse plane movements like shoulder rotation, an oblique elevated perspective improved peak range-of-motion agreement (*ICC* = 0.68). Out-of-plane tracking and fine distal rotations (forearm pronation) exhibited poor agreement (*ICC* ≤ 0.34) and substantial trajectory instability (RMSE > 30^∘^). Ultimately, while single-camera monocular RGB tracking provides sufficient validity for gross range-of-motion screening during planar upper-limb tasks, quantitative assessment of multi-planar and distal rotational kinematics requires multi-view camera fusion, depth sensing, or specialized 2D shape-regression architectures.

## 1. Introduction

Quantitative assessment of human movement is essential for evaluating motor recovery, guiding personalized therapy, and tracking longitudinal functional outcomes in individuals with neurological or musculoskeletal impairments [1,2,3]. Traditional clinical evaluations rely heavily on subjective observational scales or static goniometry, which lack dynamic spatio-temporal resolution, exhibit high inter-rater variability, and cannot capture real-time kinematic compensations during functional tasks [1,2,4,5,6]. Translating these assessments to point-of-care settings requires objective, continuous, three-dimensional (3D) motion tracking technology capable of quantifying joint range of motion, motor compensations, and longitudinal recovery trends during dynamic tasks.

To achieve such objective 3D motion analysis, rehabilitation research has historically relied on optoelectronic motion capture (Optical MoCap) systems, which represent the primary reference standard in kinematic metrology by delivering sub-millimeter spatial precision through retroreflective marker arrays [3,7,8,9]. However, Optical MoCap systems require dedicated laboratory spaces, expensive multi-camera infrastructure, physical marker attachment, and spatial calibration routines [6,8,10]. These operational burdens render traditional Optical MoCap impractical for routine point-of-care clinical assessment and impractical for unconstrained home telerehabilitation. Portable alternatives such as wearable inertial measurement units (IMUs) overcome spatial laboratory constraints, but they introduce physical sensor mounting overhead, soft-tissue artifact distortions, and cumulative drift errors over extended movement sequences [11,12,13].

To eliminate physical marker and sensor overhead while enabling scalable motion monitoring for telerehabilitation, markerless computer vision frameworks have been increasingly adapted [3,14]. Active depth-sensing cameras (e.g., Microsoft Azure Kinect) have been explored for markerless rehabilitation assessment [15], but their real-world deployment is restricted by strict operating distance windows, high computational burdens, susceptibility to ambient infrared interference, and hardware obsolescence. Multi-camera passive markerless systems (e.g., Theia3D) achieve clinical-grade biomechanical accuracy [16], yet their reliance on multi-view hardware arrays and proprietary licenses duplicates the cost and spatial complexity of traditional Optical MoCap setups [8,9]. Consequently, single-camera monocular RGB systems—requiring only a standard webcam or tablet—provide an accessible alternative for digital health and remote telerehabilitation tracking.

Recent deep learning advancements have enabled full-body skeletal tracking directly from monocular RGB video streams [17,18]. In the computer vision literature, monocular 3D human pose estimation (HPE) is predominantly approached through multi-stage 2D-to-3D lifting networks or single-stage direct regression models [19,20,21]. While temporal lifting models and large multi-person detection networks offer high modeling capacity, direct landmark regression toolboxes like Google MediaPipe have emerged as the primary choice for interactive telerehabilitation due to their cross-platform lightweight design, native CPU execution, and dual capability for global pose tracking (BlazePose) and localized hand articulation (MediaPipe Hands) [22,23]. To capture full upper-limb kinematic chains—combining gross proximal shoulder/elbow motions with fine distal forearm and hand orientations—fused pose-hand tracking pipelines have been developed [24]. However, before monocular tracking pipelines can be accepted as quantitative instruments in rehabilitation engineering and telerehabilitation, their measurement uncertainty, viewpoint dependency, and error propagation characteristics must be thoroughly validated. While computer vision benchmarks evaluate models based on 2D or 3D Euclidean landmark distance errors, rehabilitation tracking requires robust, joint angle trajectories [25,26].

Single-view 3D reconstructions suffer from inherent depth ambiguity along the camera optical axis, perspective projection distortion, and coordinate transformation sensitivities, which can induce severe trajectory drift and phase inversions in calculated joint angles [6,10,27]. Recent empirical benchmark studies have highlighted these exact degradation patterns. Balci et al. [28] evaluated monocular pose architectures against an optoelectronic standard and reported that single-camera MediaPipe 3D tracking exhibited substantially higher measurement error and lower concurrent agreement (mean ICC = 0.658) than 2D planar tracking (mean ICC = 0.848), demonstrating the vulnerability of frame-by-frame 3D depth regression. However, current validations remain constrained by critical methodological gaps: Balci et al. [28] relied on an exploratory single-subject (N = 1) design, evaluated tracking from only a single fixed perspective, omitted fine distal rotational chains (e.g., forearm pronation/supination), and evaluated monocular keypoint vectors directly without spatial coordinate normalization or alignment to a global anatomical frame. Similarly, recent work by Carnevale et al. [29] examined the influence of frontal and lateral camera configurations during musculoskeletal rehabilitation exercises, demonstrating that reconstruction accuracy is task- and viewpoint-dependent: sagittal movements showed improved agreement from a lateral view (Mean Absolute Error down to 2.7^∘^), whereas frontal views were more consistent for smaller-amplitude or multi-planar tasks (MAE 4^∘^–16^∘^). However, their evaluation was constrained to strictly orthogonal (frontal vs. lateral) planes without intermediate oblique perspectives, relied on a laboratory markerless reference rather than optoelectronic physical marker metrology, and did not examine distal multi-segment rotations such as forearm pronation. Consequently, the systematic interaction between camera viewpoint, geometric movement plane, and multi-segment coordinate transformation remains uncharacterized.

This study presents a systematic rehabilitation engineering and biomechanical metrology validation of the integrated MediaPipe pipeline [24] for monocular upper-limb kinematic tracking across a cohort of ten healthy participants (N = 10). We systematically evaluate the influence of camera perspective (frontal, oblique elevated, and anterior-oblique) across four clinical upper-limb movements representing distinct geometric movement planes: 2D planar motion (shoulder abduction), 2D transverse plane projection (shoulder internal/external rotation), 3D multi-planar motion with depth translation (elbow flexion), and fine distal multi-segment rotation (forearm pronation). By evaluating continuous trajectory tracking agreement, systematic bias, and trajectory error using identical geometric vector formulations, we define the practical measurement capabilities and viewpoint boundaries of monocular markerless motion capture for upper-limb rehabilitation assessment.

## 2. Materials and Methods

### 2.1. Measurement Cohort

Ten healthy adult males with no history of orthopedic or neurological disorders affecting the upper limbs participated in this study (mean age: 38.1 ± 11.4 years, range: 23–55 years; mean height: 1.725 ± 0.053 m; mean weight: 70.7 ± 10.6 kg; mean body mass index: 23.73 ± 3.20 kg/m^2^). All ten participants were right-handed, and all experimental movements were performed exclusively using the dominant right upper limb. The sequential order of the movement tasks was fixed across all participants. All participants received a thorough explanation regarding the study’s purpose, procedures, and potential risks, and subsequently provided written informed consent. The study protocol, including participant inclusion criteria and experimental procedures, was approved by the Ethics Committee of the Hyogo Institute of Assistive Technology (Approval Number: R2403). A sample size of ten participants is consistent with comparable biomechanical and markerless motion capture validation studies evaluating continuous multi-frame kinematic trajectories against optoelectronic instrumentation.

### 2.2. Markerless Tracking Architecture

The markerless kinematic tracking pipeline was built using the Google MediaPipe framework. To simultaneously reconstruct the gross spatial movements of the torso and arms, as well as the fine rotational mechanics of the forearm and hand, we developed the integrated MediaPipe pipeline tracking architecture [24]. This system integrates the global skeletal tracker (MediaPipe Pose) with the localized hand articulating tracker (MediaPipe Hands) in a single unified execution thread by applying a translation alignment vector at the wrist boundary to merge the localized hand coordinates into the global body space. Because MediaPipe World Landmarks express coordinates in real-world metric units and both sub-models align their local Cartesian axes (*X*,*Y*,*Z*) with the camera sensor frame, spatial fusion does not require scaling factors or inter-model rotational transformations. The hand landmarks were translated into the Pose coordinate space by matching the Hand wrist landmark to the corresponding Pose wrist landmark:

(1) $$p_{\mathrm{hand},j}^{\mathrm{fused}}=p_{\mathrm{hand},j}^{\mathrm{raw}}+\left(p_{\mathrm{wrist}}^{\mathrm{Pose}}-p_{\mathrm{wrist}}^{\mathrm{Hands}}\right)$$

where $p_{\mathrm{hand},j}^{\mathrm{raw}}$ represents the 3D position of hand landmark *j*, $p_{\mathrm{wrist}}^{\mathrm{Hands}}$ is the wrist coordinate output by MediaPipe Hands, and $p_{\mathrm{wrist}}^{\mathrm{Pose}}$ is the wrist coordinate output by MediaPipe Pose (aligned right-hand to right-wrist). While video frames exhibiting lost tracking or missing landmarks were evaluated for exclusion, the pipeline achieved a 0% frame exclusion rate across all movements and viewpoints.

**Figure 1:**
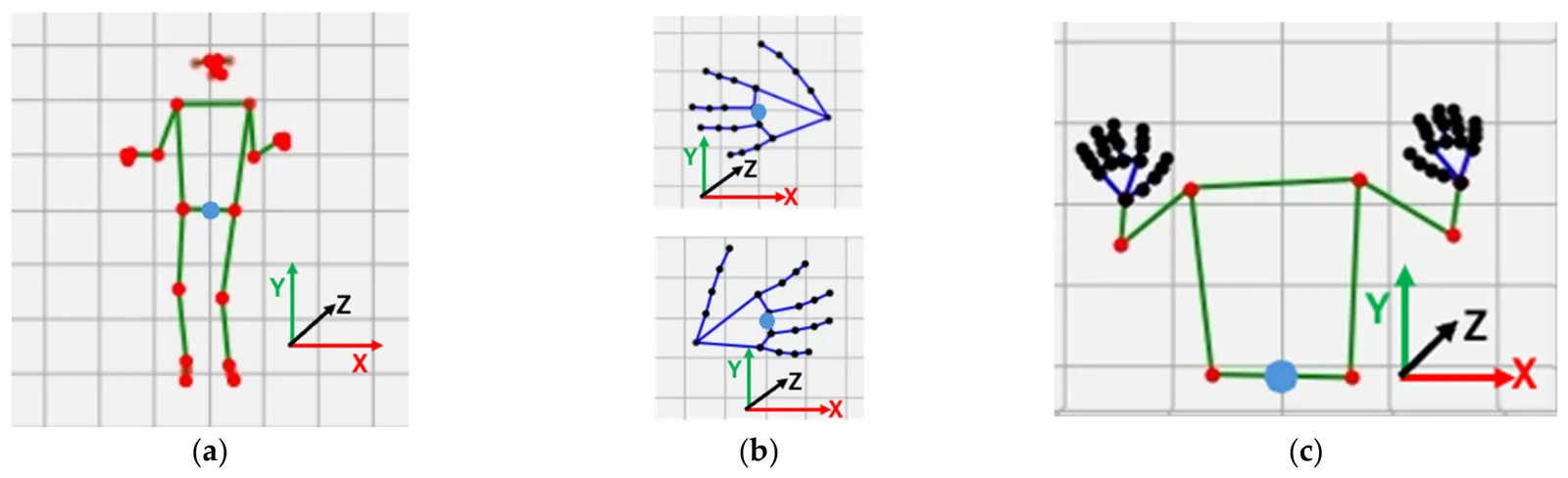
Landmark Topology of MediaPipe Pose and Hands. Blue dot = Origin of Landmarks: (**a**) Landmarks detected by MediaPipe Pose. (**b**) Landmarks detected by MediaPipe Hands. (**c**) Sample of Combined landmarks of Hands and Pose.

To prioritize measurement resolution over computational execution speed, we selected the highest model complexity configurations within the MediaPipe API: Complexity Level 2 for the Pose model, and Complexity Level 1 for the Hands model. Both detection and tracking confidence thresholds were set to 0.5 (min_detection_confidence = 0.5, min_tracking_confidence = 0.5). Internal landmark temporal smoothing was disabled (smooth_landmarks = False) to prevent artificial filtering and assess the raw spatial inference capability of the network. Kinematic vectors were calculated using MediaPipe’s “World Landmarks” coordinate output array. Data processing was conducted using MediaPipe version 0.8.10 in Python 3.11 on an Apple M1 MacBook Pro running macOS. This database provides 3D spatial coordinates in a metric scale relative to a local origin defined at the subject’s hip center (Figure 1).

### 2.3. Experimental Design and Metrology Setup

**Figure 2:**
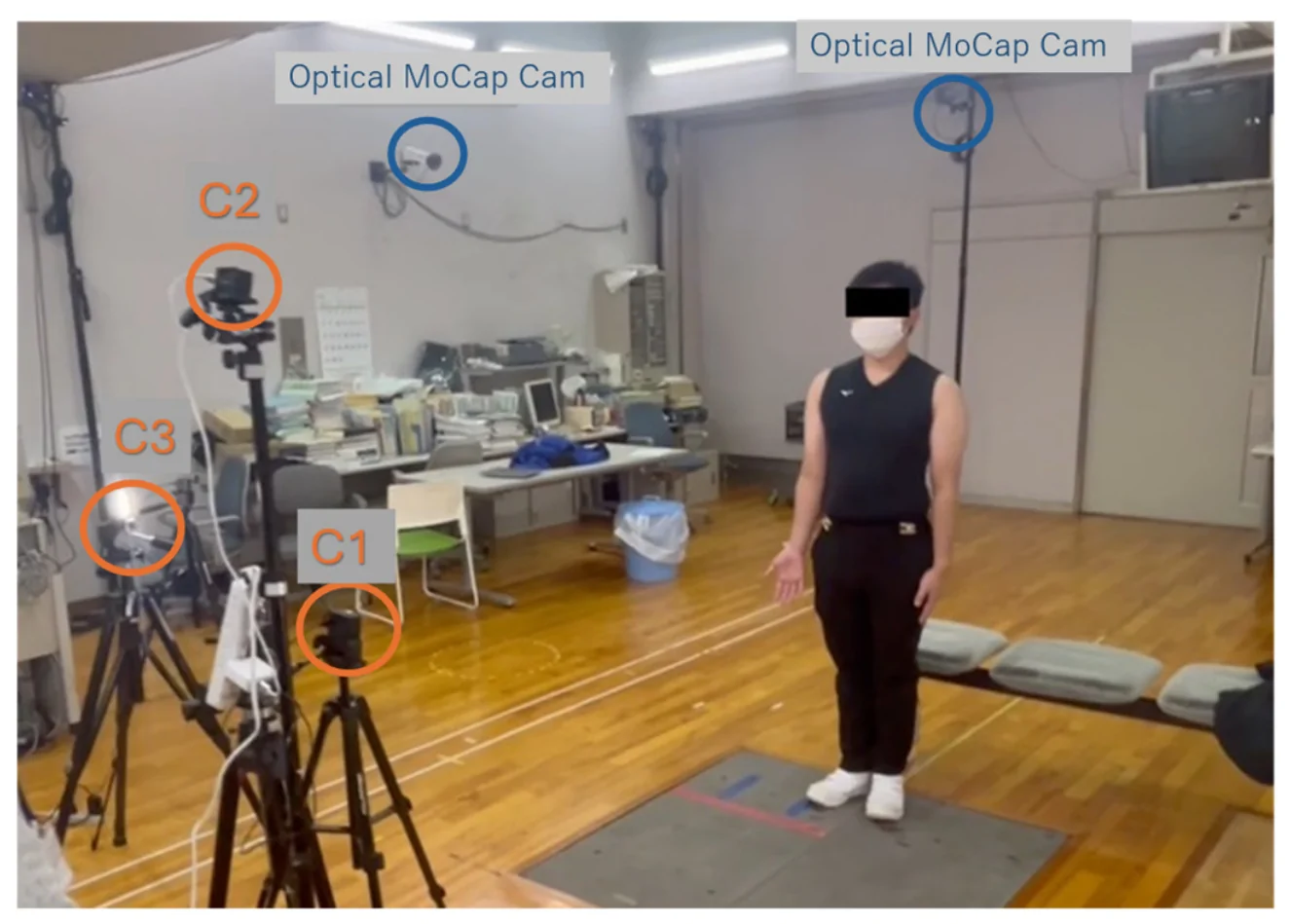
The Experimental Setup.

**Figure 3:**
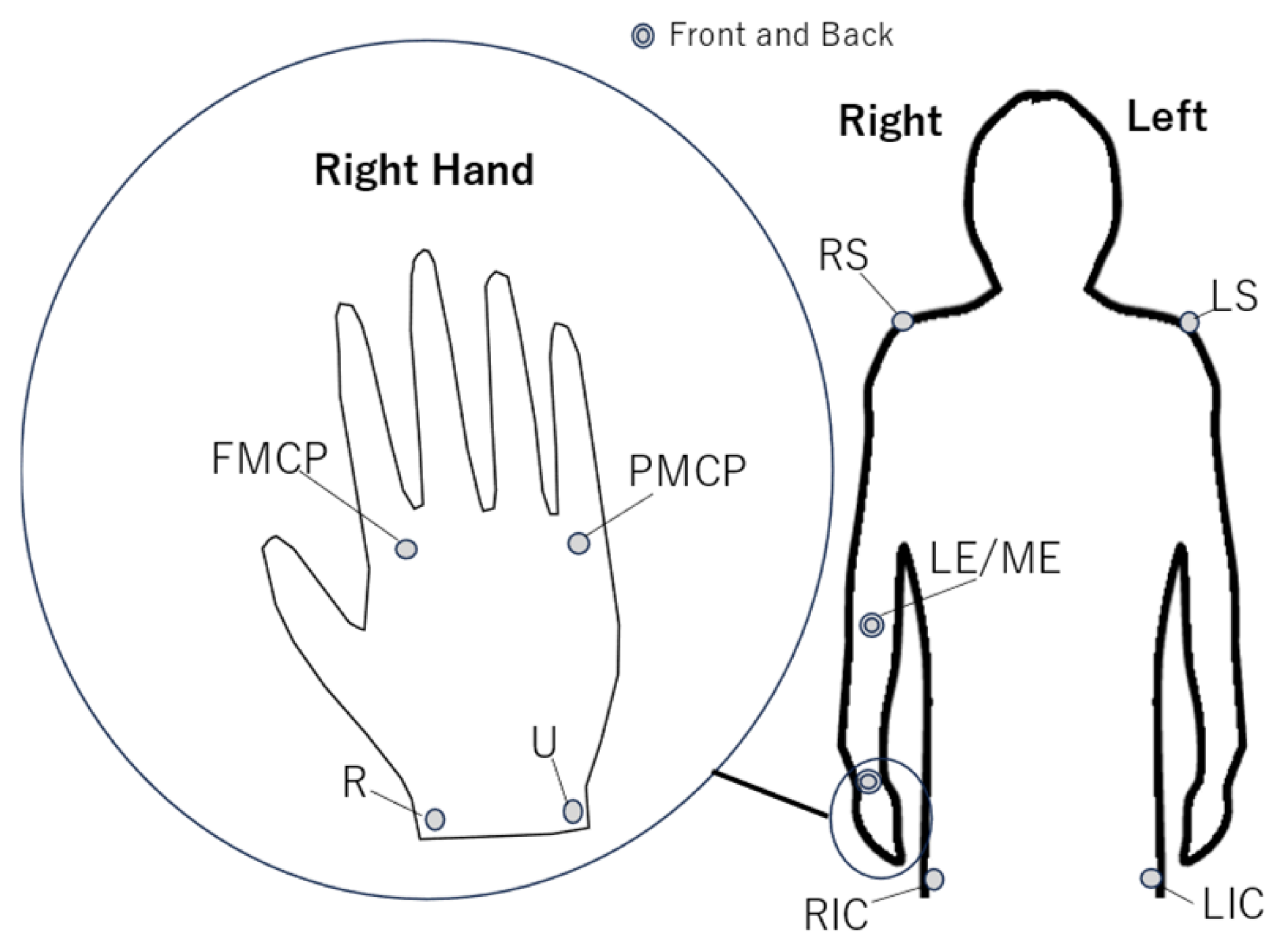
The locations of reflective marker placement. The surface markers are abbreviated as follows: RS (Right Shoulder), LS (Left Shoulder), RIC (Right Iliac Crest), LIC (Left Iliac Crest), LE (Lateral Epicondyle), ME (Medial Epicondyle), R (Radial Styloid), U (Ulnar Styloid), FMCP (2nd Metacarpophalangeal Joint), and PMCP (5th Metacarpophalangeal Joint).

To establish the reference kinematic baseline, an 8-camera optoelectronic system (Vicon, Oxford Metrics, Oxford, UK) was used to record physical retroreflective marker positions at a sampling frequency of 100 Hz (Figure 2). Spherical retroreflective markers (14 mm diameter) were placed as shown in Figure 3. Standard optoelectronic motion capture protocols typically rely on proprietary biomechanical models (e.g., Vicon Plug-in Gait) to estimate internal joint centers from surface markers. However, applying different mathematical models to the two systems would introduce confounding algorithmic biases. Therefore, to isolate and evaluate the raw spatial tracking performance of the MediaPipe algorithm, we intentionally bypassed optoelectronic joint-center modeling. Instead, we applied the identical mathematical vector formulations (Table 1) to both the MediaPipe inferred landmarks and the raw optoelectronic surface marker coordinates. To establish parity with MediaPipe’s single-point outputs, the optoelectronic shoulder and metacarpophalangeal (MCP) nodes were represented directly by their raw surface, while the elbow and wrist coordinates were calculated as the 3D spatial midpoints of their respective paired markers (medial and lateral epicondyles; radial and ulnar styloids). This midpoint calculation was strictly necessary to preserve stable longitudinal spatial vectors for the arm segments. Specifically, because the radial and ulnar surface markers move with the palm during pronation and supination, relying on a single distal marker would artificially shift the spatial direction of the elbow-to-wrist vector depending on the hand’s orientation. Calculating the 3D midpoint neutralizes this displacement, yielding a consistent central axis regardless of palm direction. Because the shoulder and metacarpophalangeal (MCP) nodes do not experience this specific vector-displacing orbital rotation, midpoint calculations were unnecessary for these locations. Therefore, they were represented directly by their raw single surface coordinates to mirror the markerless topology. Consequently, this study evaluates direct tracking geometry rather than clinically modeled joint centers. In Vicon Nexus, any brief marker trajectory dropouts were filled using cubic spline interpolation, and the raw 100 Hz marker trajectories were low-pass filtered using a zero-phase fourth-order Butterworth filter with a 6 Hz cutoff frequency, conforming to clinical biomechanical standards.

**Figure 4:**
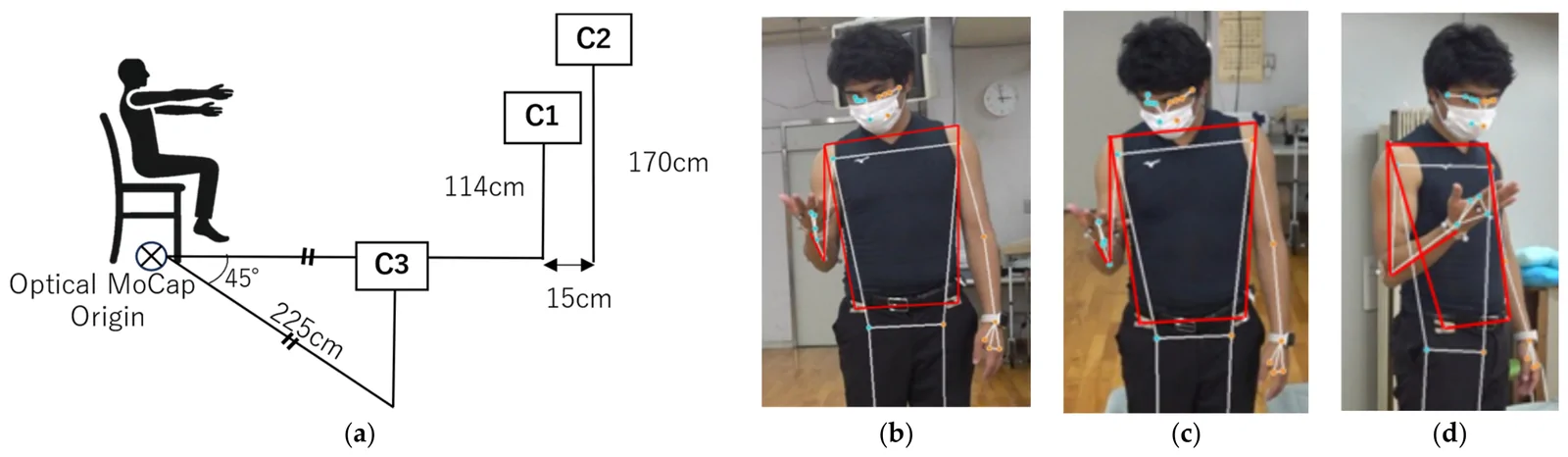
Experimental camera configuration and corresponding multi-view spatial vector tracking. (**a**) Schematic layout detailing the spatial positions of the three RGB cameras (C1, C2, and C3) relative to the subject. (**b**–**d**) Representative fields of view from C1, C2, and C3, respectively, illustrating the simultaneous tracking overlays. The white lines represent the pre-defined spatial landmarks inferred by the MediaPipe algorithm. Blue dot and orange dot represent right side and left side of the body, respectively. The red lines represent the direct geometric tracking vectors derived from the optoelectronic surface markers. Note: C stands for Camera.

The monocular validation setup consisted of three RGB cameras (Sony RX0, Sony Corp., Tokyo, Japan) recording at a resolution of 1920 × 1080 pixels and a frame rate of 60 fps. The cameras operated with a fixed 24 mm equivalent focal length (f/4.0 ZEISS Tessar T lens) under factory optical settings without additional lens distortion correction, and all recorded video streams were processed at their native 1080p resolution without downscaling. To study viewpoint dependency, the cameras were placed in three distinct spatial positions (Figure 4):

- Camera 1 (C1) was placed directly in front of the subject at eye level (114 cm height) to capture the frontal projection plane.
- Camera 2 (C2) was positioned at an oblique, elevated perspective (170 cm height, tilted downward) to capture 3D coordinate shifts.
- Camera 3 (C3) was placed at an oblique angle (45° offset relative to the sagittal plane, 114 cm height) to capture spatial depth transitions.

Because monocular anterior cameras inherently suffer from depth ambiguity along their optical axis during movements with anterior–posterior trajectories, Camera 2 (elevated) and Camera 3 (45° offset) were strategically deployed to project these out-of-plane kinematic displacements onto orthogonal and oblique 2D image planes, thereby enhancing keypoint localization.

To evaluate performance across different motion geometries, subjects performed four single-joint upper-limb movements representing distinct degrees of freedom and spatial tracking challenges:

- Shoulder Abduction: Standing task, representing relative segment-to-segment tracking on the 2D frontal plane.
- Shoulder Rotation (Internal/External): Seated task with the arm supported, representing rotation on the 2D transverse plane.
- Elbow Flexion: Standing task, representing dynamic 3D multi-planar tracking with substantial depth changes.
- Forearm Pronation: Seated task, representing a high-resolution, multi-segment rotation tracking problem. To isolate radioulnar rotation and prevent kinematic confounding, the upper limb posture was strictly constrained: the arm was held in a fixed position with the palm anatomically aligned with the shoulder axis. Pronation was executed solely by rotating the palm, ensuring the elbow flexion angle remained static throughout the motion.

For each movement, subjects initiated the trial from a rest state, progressively increasing the range of motion across three continuous repetitions.

The four upper-limb movements were specifically selected to represent fundamental clinical degrees of freedom, and their joint angles were calculated in accordance with the standard range-of-motion measurement guidelines established by the Japanese Orthopaedic Association (JOA) and the Japanese Association of Rehabilitation Medicine (JARM) [30,31].

### 2.4. Spatio-Temporal Data Alignment

Prior to temporal alignment, the raw coordinate trajectories from the webcams were passed through a zero-phase, fourth-order Butterworth low-pass filter with a cutoff frequency of 3 Hz to eliminate high-frequency tracking jitter without introducing temporal lag [24]. To resolve the temporal mismatch between the Optical MoCap system (100 Hz) and the webcams (60 fps), the Optical MoCap coordinate trajectories were downsampled to 60 Hz using a linear interpolation algorithm.

Temporal synchronization between the two independent recording systems was performed via cross-correlation analysis. Specifically, the cross-correlation function was computed between the kinematic angle waveforms of the shoulder abduction task for each participant and camera viewpoint. Because each participant performed the entire experimental sequence continuously without interruption, the single calculated lag offset corresponding to the peak cross-correlation was applied uniformly to align the entire continuous time-series recording for that participant and camera condition.

### 2.5. Virtual Frontalization Coordinate Transformation

To apply consistent joint-angle formulations across all three camera viewpoints, we implemented a coordinate transformation framework (virtual frontalization) prior to kinematic angle calculation, standardizing the landmarks to an anterior perspective irrespective of the original recording angle. This mathematical framework transforms the raw coordinate array $p_{i}^{\mathrm{raw}}={\left[x_{i}^{\mathrm{raw}},y_{i}^{\mathrm{raw}},z_{i}^{\mathrm{raw}}\right]}^{T}$ of each tracked joint landmark *i* into a local, subject-centered coordinate system aligned with the pelvis anatomical plane. This step neutralizes the physical offset of the camera placement.

Let ***p***_R.Hip_ and ***p***_L.Hip_ be the spatial positions of the right and left hip centers, respectively. The lateral coordinate axis vector ***u****_x_* is defined as:

(2) $$u_{x}=\frac{p_{R.\mathrm{Hip}}-p_{L.\mathrm{Hip}}}{∥p_{R.\mathrm{Hip}}-p_{L.\mathrm{Hip}}∥}$$

Let *p*_Trunk_ be the midpoint of the shoulder centers, and *p*_Pelvis_ be the midpoint of the hip centers:

(3) $$p_{\mathrm{Trunk}}=\frac{1}{2}\left(p_{R.\mathrm{Shoulder}}+p_{L.\mathrm{Shoulder}}\right),p_{\mathrm{Pelvis}}=\frac{1}{2}\left(p_{R.\mathrm{Hip}}+p_{L.\mathrm{Hip}}\right)$$

The temporary vertical axis vector is defined as $u_{y}^{\prime }=p_{\mathrm{Trunk}}-p_{\mathrm{Pelvis}}$. The orthogonal depth axis vector *u_z_* is then calculated as:

(4) $$u_{z}=\frac{u_{x}\times u_{y}^{\prime }}{∥u_{x}\times u_{y}^{\prime }∥}$$

The true vertical axis vector is defined as *u_y_* = *u_z_* × *u_x_*. Using these vectors, we construct the rotation transformation matrix $R={\left[u_{x},u_{y},u_{z}\right]}^{T}$. Any raw joint coordinate is then mapped to its normalized position $p_{i}^{\mathrm{frontal}}$ in the virtual frontal plane using:

(5) $$p_{i}^{\mathrm{frontal}}=R\left(p_{i}^{\mathrm{raw}}-p_{\mathrm{Pelvis}}\right)$$

All subsequent 2D and 3D kinematic angle calculations for both markerless and optoelectronic datasets were performed strictly using these transformed, body-fixed coordinates, ensuring that trunk orientation relative to the camera does not confound joint angle calculations.

### 2.6. Kinematic Formulations

Table 1 summarizes the spatial definitions of the joint segment angles computed using the transformed coordinates.

The kinematic computations are formulated as follows:

- 2D Planar Angles: Projected directly onto specified segment planes:

*θ*_2*D*_ = (*atan*2(*y*_1_,*x*_1_) − *atan*2(*y*_2_,*x*_2_)) (6)

- 3D Multi-Segment Angles: Derived from the dot product of relative segment spatial vectors:

(7) $$\theta_{3D}=\arccos\left(\frac{v_{1}{\cdot v}_{2}}{\left|v_{1}\right|\left|v_{2}\right|}\right)$$

- Forearm Rotations: Determined by computing the normal vector ***n***_hand_ of the palm plane (defined by the wrist center, 2nd metacarpophalangeal [MCP], and 5th MCP joints) relative to the upper arm anatomical vector ***v***_arm_:

(8) $$\theta_{\mathrm{plane}}=90{}^{\circ}-\arccos\left(\frac{n_{\mathrm{hand}}\cdot v_{\mathrm{arm}}}{\left|n_{\mathrm{hand}}\right|\left|v_{\mathrm{arm}}\right|}\right)$$

### 2.7. Statistical Metrics

Peak Range-of-Motion Agreement (Intraclass Correlation Coefficient): To ensure statistical independence, peak range of motion (ROM) was extracted for each participant, task, and camera viewpoint. The Intraclass Correlation Coefficient (*ICC*_2.1_, two-way random-effects model, single measures, absolute agreement) was computed on these participant-level summary metrics (*n* = 10 subjects, *k* = 2 measurement systems) [32].

(9) $$\mathrm{IC}C_{2,1}=\frac{{\mathrm{MS}}_{R}-{\mathrm{MS}}_{E}}{{\mathrm{MS}}_{R}+\left(k-1\right){\mathrm{MS}}_{E}+\frac{k}{n}({\mathrm{MS}}_{C}-{\mathrm{MS}}_{E})}$$

where *MS_R_* is the mean square for rows (participants); *MS_E_* is the mean square for error; *MS_C_* is the mean square for columns (measurement systems, *k* = 2); and *n* represents the total number of participants (*n* = 10). To account for the small sample size, 95% confidence intervals were derived using non-parametric bootstrap resampling (2000 iterations). ICC values were interpreted according to Koo and Li [32]: <0.50 (poor), 0.50–0.75 (moderate), 0.75–0.90 (good), and >0.90 (excellent).

Trajectory Tracking Accuracy (Root Mean Square Error): To quantify overall time-series waveform agreement and cumulative tracking deviation across entire movement cycles, the Root Mean Square Error (RMSE) was calculated for each kinematic waveform relative to the optoelectronic reference:

(10) $$\mathrm{RMSE}=\sqrt{\frac{1}{N}\sum_{i=1}^{N}{\left(\theta_{\mathrm{makrkerless}}\left(i\right)-\theta_{\mathrm{Optical}\;\mathrm{MoCap}}\left(i\right)\right)}^{2}}$$

Systematic Bias and Limits of Agreement: Inferential Bland–Altman systematic bias (*d*) and 95% limits of agreement (LoA = *d* ± 1.96 × *SD*_diff_) were calculated using participant-level peak ROM differences (*N* = 10). A two-tailed one-sample *t*-test (*df* = 9) was conducted to evaluate whether the mean systematic bias differed significantly from zero (*p* < 0.05), with exact *p*-values and 95% confidence intervals reported.

Visual Inspection: Across all 12 experimental conditions (4 movement tasks × 3 camera views), coordinate trajectories from both systems were visually inspected to assess shape and amplitude consistency, persistent offsets, and transient tracking instability, such as spurious tracking spikes or complete joint tracking failures.

## 3. Results

**Figure 5:**
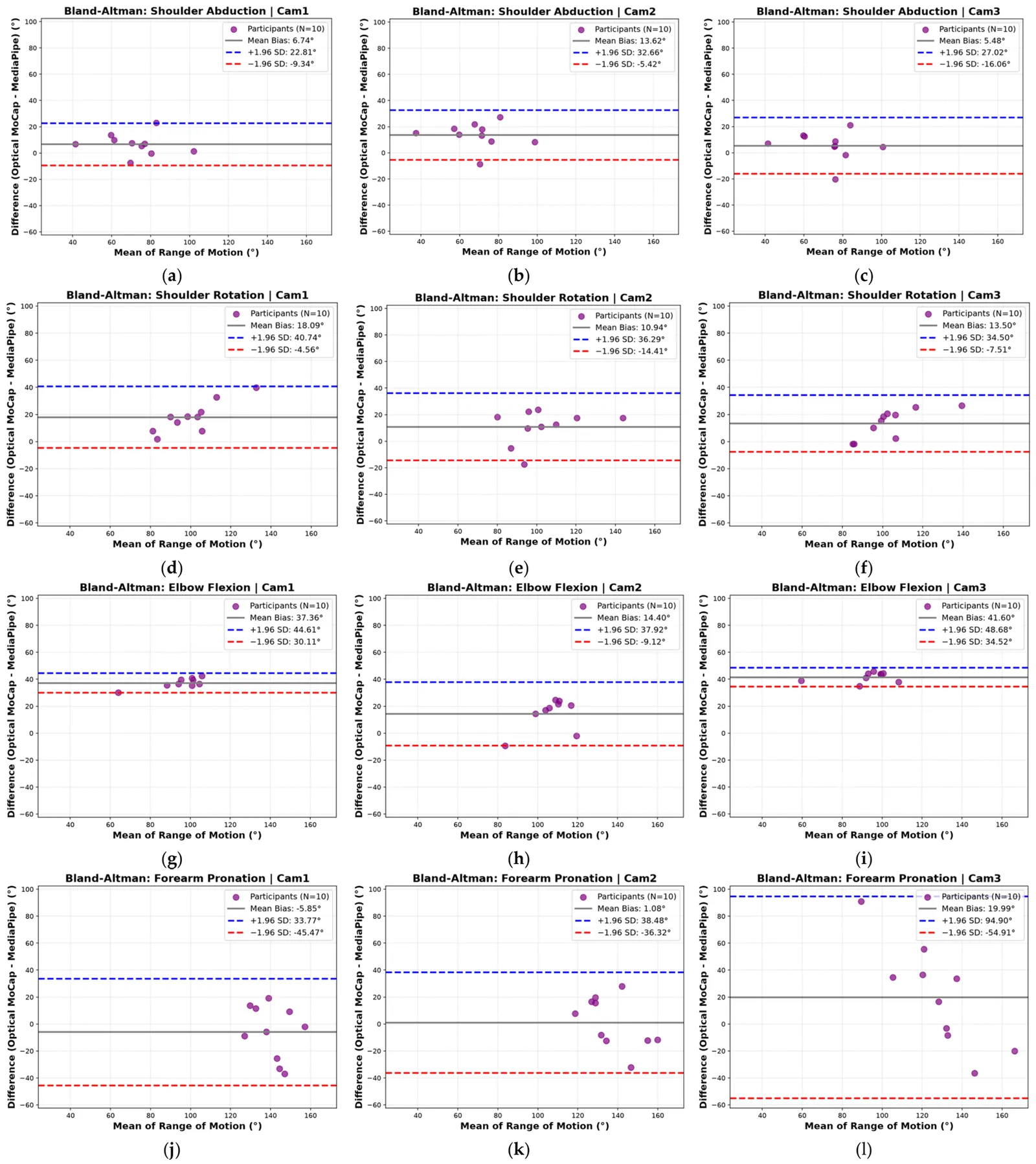
Bland–Altman agreement analysis across all participants (*N* = 10) comparing monocular MediaPipe RGB tracking against the optoelectronic reference system across 12 experimental conditions (4 movement tasks × 3 camera viewpoints). The solid horizontal line indicates the mean systematic bias (°); blue and red dashed horizontal line represent the upper and lower 95% limits of agreement, respectively (LoA = Bias ± 1.96 SD): (**a**) Camera 1—Shoulder abduction, (**b**) Camera 2—Shoulder abduction, (**c**) Camera 3—Shoulder abduction, (**d**) Camera 1—Shoulder rotation, (**e**) Camera 2—Shoulder rotation, (**f**) Camera 3—Shoulder rotation, (**g**) Camera 1—Elbow flexion, (**h**) Camera 2—Elbow flexion, (**i**) Camera 3—Elbow flexion, (**j**) Camera 1—Forearm pronation, (**k**) Camera 2—Forearm pronation, and (**l**) Camera 3—Forearm pronation.

**Figure 6:**
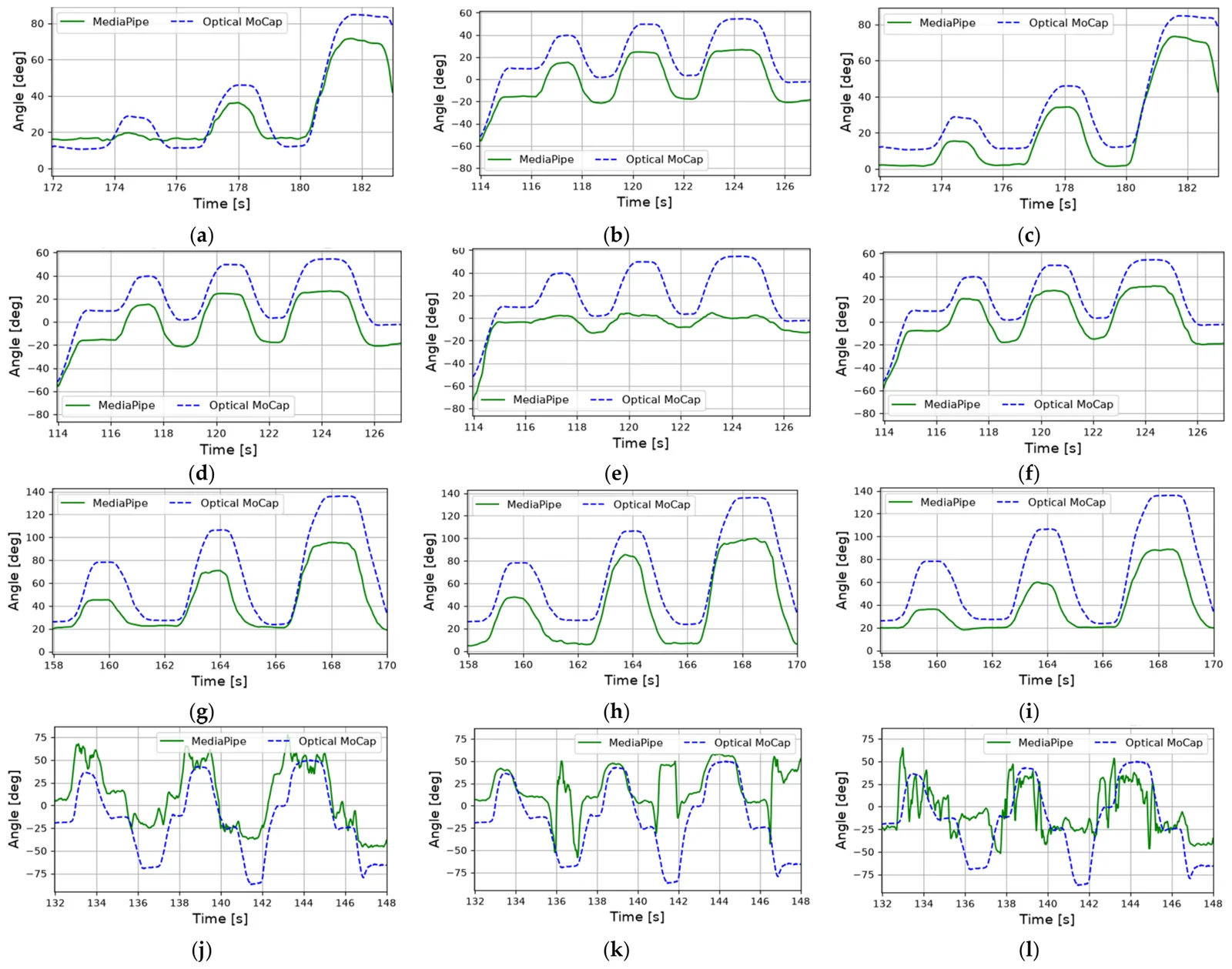
Time-series joint-angle trajectories from both tracking systems: (**a**) Camera 1—Shoulder abduction, (**b**) Camera 2—Shoulder abduction, (**c**) Camera 3—Shoulder abduction, (**d**) Camera 1—Shoulder rotation, (**e**) Camera 2—Shoulder rotation, (**f**) Camera 3—Shoulder rotation, (**g**) Camera 1—Elbow flexion, (**h**) Camera 2—Elbow flexion, (**i**) Camera 3—Elbow flexion, (**j**) Camera 1—Forearm pronation, (**k**) Camera 2—Forearm pronation, and (**l**) Camera 3—Forearm pronation.

The kinematic tracking outcomes, trajectory agreement coefficients, and error metrics across all tested camera viewpoints and joint geometries are summarized in Table 2. All kinematic angle trajectories presented in the subsequent agreement and waveform analyses (Figure 5 and Figure 6) were calculated according to the planar and 3D definitions established in Table 1, utilizing the normalized coordinates derived from the virtual frontalization transformation.

### 3.1. Peak Range-of-Motion Agreement and Viewpoint Sensitivity

The peak range-of-motion agreement of the monocular tracking system was highly dependent on the viewpoint of the camera and the plane of movement:

- Shoulder Abduction (Planar, Relative Angle): Demonstrated moderate-to-good agreement across all viewpoints (*ICC*_2.1_ = 0.63–0.82). The highest coefficient was achieved under Camera 1 (*ICC*_2.1_ = 0.82 [95% CI: 0.33, 0.95]), followed by Camera 3 (*ICC*_2.1_ = 0.77 [95% CI: 0.12, 0.93]) and Camera 2 (*ICC*_2.1_ = 0.63 [95% CI: 0.07, 0.83]).
- Shoulder Rotation (Out-of-Plane, Transverse Projection): Exhibited moderate agreement from the oblique viewpoints (Camera 2: *ICC*_2.1_ = 0.68 [95% CI: 0.04, 0.83]; Camera 3: *ICC*_2.1_ = 0.60 [95% CI: 0.18, 0.76]), but declined to poor agreement under the direct frontal view (Camera 1: *ICC*_2.1_ = 0.47 [95% CI: 0.17, 0.60]).
- Elbow Flexion (Out-of-Plane, 3D Angle): Exhibited poor agreement across all perspectives (*ICC*_2.1_ = 0.18–0.31; Camera 1: 0.19 [95% CI: 0.01, 0.35]; Camera 2: 0.31 [95% CI: 0.01, 0.61]; Camera 3: 0.18 [95% CI: 0.01, 0.33]).
- Forearm Pronation (Segment Fusion): Failed to achieve acceptable agreement across all camera views (*ICC*_2.1_ = −0.07–0.34; Camera 1: −0.07 [95% CI: −0.56, 0.57]; Camera 2: 0.34 [95% CI: −0.27, 0.70]; Camera 3: 0.09 [95% CI: −0.29, 0.39]).

### 3.2. Trajectory Tracking Accuracy (RMSE)

Trajectory tracking accuracy was strongly governed by movement plane geometry. Planar shoulder abduction achieved the lowest trajectory tracking error across all viewpoints (RMSE = 9.00^∘^–11.23^∘^), with Camera 2 achieving the lowest error (RMSE = 9.00^∘^), followed by Camera 1 (RMSE = 11.10^∘^) and Camera 3 (RMSE = 11.23^∘^).

In contrast, tasks with substantial out-of-plane depth components or distal rotations demonstrated considerably larger trajectory errors: shoulder rotation yielded RMSE values between 17.68^∘^ and 22.52^∘^ (Camera 2: 17.68^∘^, Camera 3: 18.06^∘^, Camera 1: 22.52^∘^). Elbow flexion tracking errors ranged from 23.75^∘^ to 30.92^∘^ (Camera 1: 23.75^∘^, Camera 2: 24.63^∘^, Camera 3: 30.92^∘^). Forearm pronation exhibited the greatest trajectory deviation, with RMSE values exceeding 30^∘^ across all viewpoints (Camera 1: 30.11^∘^, Camera 3: 34.74^∘^, Camera 2: 45.69^∘^).

### 3.3. Systematic Biases

The systematic bias varied across the joint segments (Table 2). For shoulder abduction, Camera 3 showed a small, non-significant bias of 5.48^∘^ (*p* = 0.149), whereas Camera 1 and Camera 2 exhibited positive biases of 6.75^∘^ (*p* = 0.029) and 13.62^∘^ (*p* = 0.002). Shoulder rotation displayed consistent positive systematic bias across all viewpoints, peaking at 18.09^∘^ (*p* = 0.001) under Camera 1. Similarly, elbow flexion exhibited significant positive systematic bias across all perspectives (+14.40^∘^ to +41.60^∘^, all *p* ≤ 0.007), reflecting constant joint angle overestimation. Conversely, forearm pronation demonstrated non-significant systematic bias across all camera views (−5.71^∘^ to 19.99^∘^, all *p* ≥ 0.132), although accompanied by marked expansion of the limits of agreement.

### 3.4. Bland–Altman Agreement of Peak Range of Motion

Bland–Altman analysis of participant-level peak range of motion (Figure 5) showed varied agreement depending on the movement task. Shoulder abduction (Figure 5a–c) displayed differences centered near the zero-error line, indicating low systematic bias. In contrast, elbow flexion (Figure 5g–i) exhibited the narrowest limits of agreement (tightest data clustering), but the data points were shifted predominantly above zero. This large positive bias suggests a consistent systematic offset between the optoelectronic marker-based reference and the markerless landmark estimations. Shoulder rotation (Figure 5d–f) displayed a similar, though less pronounced, positive bias. Finally, forearm pronation (Figure 5j–l) exhibited high vertical scatter. This wide dispersion resulted in broad limits of agreement (e.g., spanning >130° in Camera 3), illustrating the reduced precision of monocular tracking for complex distal axial rotations.

### 3.5. Visual Waveform Inspection

Figure 6 presents representative time-series joint angle trajectories comparing the monocular and optoelectronic tracking systems across the 12 conditions for a representative participant (Participant 9, whose trajectories exhibited median tracking error across tasks).

These waveforms reveal several tracking trends:

- Amplitude and Phase Alignment: Highly planar tracking tasks (shoulder abduction; Figure 6a–c) show excellent alignment in both amplitude and phase across all three viewpoints.
- Systematic Offsets: Elbow flexion (Figure 6g–i) shows high phase correlation but maintains a constant, systematic offset throughout the range of motion.
- Tracking Failures and Inversions: Forearm pronation (Figure 6j–l) exhibits severe tracking instability, characterized by sudden trajectory jumps, high-frequency noise, and phase inversion artifacts.

## 4. Discussion

This study evaluated the concurrent validity and inter-system agreement of an integrated monocular markerless motion-tracking pipeline against an optoelectronic reference system across four upper-limb movements and three camera viewpoints. By analyzing participant-level peak range of motion (*N* = 10) alongside continuous trajectory errors (RMSE), our findings demonstrate that monocular tracking performance depends heavily on the geometric relationship between the movement plane and the camera optical axis. This interpretation is supported by Francia et al. [3], who reported that tracking accuracy is strongly influenced by camera perspective, self-occlusion, and anatomical joint geometry.

### 4.1. Perspective Projection Mechanics and Dimensional Vulnerability

Planar joint kinematics moving parallel to the camera sensor plane—specifically shoulder abduction—achieved the highest agreement (*ICC*_2.1_ = 0.63–0.82) and lowest trajectory errors (RMSE = 9.00^∘^–11.23^∘^). Because the upper arm moves within the 2D image plane, the neural network directly detects large pixel displacements without relying on depth inference.

In contrast, joint kinematics with significant out-of-plane components showed marked performance degradation and strong viewpoint sensitivity. Shoulder internal/external rotation maps directly along the camera optical axis from the frontal perspective (Camera 1), resulting in poor peak ROM agreement (*ICC*_2.1_ = 0.47) and elevated trajectory error (RMSE = 22.52^∘^). Because monocular vision cannot directly measure depth, coordinates along the optical axis are estimated probabilistically, introducing substantial uncertainty. This depth-axis vulnerability directly mirrors observations by Balci et al. [28], who reported that single-camera MediaPipe 3D tracking yielded substantially lower agreement than 2D planar tracking when validated against an optoelectronic system, as well as systematic review evidence by Lee et al. [6] documenting reduced validity for rotational shoulder kinematics. When the camera is repositioned to an elevated or oblique perspective (Cameras 2 and 3), part of this rotational displacement projects onto the lateral image plane, improving agreement (*ICC*_2.1_ = 0.60–0.68) and reducing error (RMSE = 17.68^∘^–18.06^∘^). Specifically, the elevated perspective (Camera 2) achieved the highest agreement, suggesting that tracking improves as the anatomical plane of motion aligns more closely with the camera sensor plane. These results align with findings by Carnevale et al. [29], who demonstrated that markerless tracking error during rehabilitation tasks depends on viewing perspective, with lateral configurations outperforming frontal views for sagittal movements.

### 4.2. Anatomical Coordinate System Sensitivity and Error Propagation

In clinical motion analysis and rehabilitation biomechanics, acceptable error thresholds depend heavily on the intended application. In laboratory gait and formal diagnostic kinematic analysis, errors below 5^∘^ are typically demanded to distinguish subtle pathological deviations. Conversely, for gross functional assessment, remote telerehabilitation screening, and clinical range-of-motion tracking, errors within 5^∘^–10^∘^ are widely considered acceptable, especially given that Minimum Clinical Difference (MCD) for manual goniometry ranges from 11° to 16° for a single clinician, and 14° to 24° between multiple clinicians [33]. In the present study, shoulder abduction was the only task that yielded trajectory tracking errors near this range (RMSE = 9.00–11.23°). However, the systematic bias for this task ranged from 5.48° to 13.62° depending on the camera viewpoint. All other tasks substantially exceeded the 10° threshold, with RMSE values for shoulder rotation (17.68–22.52°), elbow flexion (23.75–30.92°), and forearm pronation (30.11–45.69°) demonstrating severe trajectory deviations. These findings suggest that unassisted monocular tracking under these specific conditions does not currently provide sufficient precision for the quantitative assessment of multi-planar or rotational upper-limb kinematics.

The large systematic biases observed in elbow flexion (+14.40° to +41.60°, *p* < 0.007) appear to be associated with a structural measurement discrepancy inherent to our experimental design. While previous validation studies often utilize proprietary optoelectronic software to map surface markers to internal joint centers prior to comparison [3], our methodology intentionally calculated angles directly from raw surface marker trajectories to ensure identical kinematic vector formulations between the two systems. Because BlazePose probabilistically infers generalized structural keypoints (based on human-annotated training data) while our optoelectronic baseline utilized a combination of raw surface coordinates (at the shoulder) and simple spatial midpoints (at the elbow/wrist), a constant, systematic anatomical offset remains highly visible at the proximal shoulder joint. This offset highlights a critical methodological trade-off: while utilizing unmodeled surface geometry and spatial midpoints successfully stabilizes rotational axes without requiring complex proprietary biomechanical modeling, it inherently diverges from the probabilistic spatial landmarks generated by the AI. Therefore, when validating markerless systems, researchers must strictly account for the spatial discrepancy between physical surface marker geometry and the AI’s inferred topological network.

### 4.3. The Pose-Hand Interface Boundary Problem

The severe tracking failures and phase inversions observed in forearm pronation (ICC = −0.08–0.34, RMSE > 30° in oblique views) appear to be associated with architectural limitations in the deep-learning pipeline. Because the global Pose architecture lacks the resolution for fine-grained hand mechanics, the pipeline extracts a localized bounding box around the wrist joint and passes this image to the Hands sub-model. While recent benchmarks by Balci et al. [28] omitted distal rotational chains, our fused Pose–Hand evaluation highlights two critical vulnerabilities at this tracking boundary:

- Temporal and Spatial Discontinuity: The localized Hand crop is processed as an isolated 2D image. This ignores the anatomical continuity and mechanical constraints of the full arm (e.g., the kinematic link between the shoulder, elbow, and wrist).
- Palmar-Dorsal Topology Flipping: Visual trajectory inspection suggests that as the hand rotates, it passes through an intermediate orientation where the palm plane is nearly parallel to the camera viewing vector. In this orientation, the Hands model appears to intermittently misidentify the palmar and dorsal surfaces, leading to sudden topology reversals and phase jumps in calculated palm normal angles (Figure 6k,l). While systematic failure-rate classification was not performed, this observation highlights the fragility of unconstrained monocular depth inference during axial limb rotations.

To overcome these depth-reconstruction failures, recent studies have proposed data-driven regression models (such as LightGBM) that predict joint angles using only the highly reliable 2D image coordinates [5]. By analyzing 2D shape features (such as the changing area of the triangle formed by the wrist, index, and pinky landmarks), these models bypass the unstable 3D coordinate estimation step, achieving forearm tracking errors as low as 4.65^∘^ − 5.61^∘^. This suggests that for monocular systems, data-driven 2D geometric regressions are significantly more robust than direct calculations based on estimated 3D coordinates.

### 4.4. Engineering Guidelines for Monocular Markerless Motion Tracking

To guide the design of monocular kinematic measurement systems utilizing anterior-facing camera setups, we establish an engineering classification map (Table 3) based on movement geometry and tracking tolerances.

This categorization defines the practical limits of monocular tracking. For gross planar tracking tasks (such as shoulder abduction), the monocular system demonstrates technical potential to approximate gross range of motion and identify substantial compensatory movements exceeding 15^∘^ [15]. However, the observed trajectory error (RMSE ≈ 9.00^∘^–11.23^∘^) indicates that monocular RGB tracking is currently restricted to coarse screening rather than fine diagnostic assessment. Future studies must directly evaluate these tracking boundaries in clinical cohorts exhibiting atypical motor synergies and joint contractures before clinical implementation can be established.

Similarly, given that variation (MCD) in manual goniometric measurements of shoulder abduction specifically can reach 16° for a single clinician and up to 20°–24° between multiple clinicians [33], our system’s measurement precision for shoulder abduction (RMSE = 9.00–11.23°) suggests potential utility for gross longitudinal tracking. However, for precise assessment of out-of-plane movements (such as shoulder rotation, elbow flexion, or forearm pronation), current monocular tracking algorithms are inadequate. For these applications, systems must integrate multi-camera arrays [3,14], depth sensors, or customized 2D machine-learning models to achieve high diagnostic precision.

### 4.5. System Constraints and Research Directions

Several limitations must be addressed in future work:

I. Cohort Homogeneity: The validation cohort was restricted to a small sample (N = 10) of healthy subjects with normal joint mechanics and body mass indices. In actual clinical applications, individuals with neurological or orthopedic impairments often exhibit atypical joint mechanics, contractures, and physical mobility aids that may challenge computer-vision landmark detection. Future studies must evaluate tracking robustness under these diverse clinical conditions.
II. Temporal Cross-Correlation Synchronization: Temporal synchronization relied on cross-correlation alignment applied to the shoulder abduction task. While this effectively corrected fixed clock offsets between the two independent recording systems, cross-correlation alignment may partially absorb intrinsic system response latencies, thereby presenting an optimistic estimate of dynamic phase agreement.
III. Controlled Environment: This validation was conducted in a controlled laboratory setting with optimized lighting and static backgrounds. Real-world environments (such as clinical wards or home telerehabilitation settings) introduce challenging conditions, including backlighting, dynamic backgrounds, and visual occlusions.
IV. Single-Camera Depth Constraints: Single-camera systems present fundamental physical limitations, particularly the loss of depth information along the optical axis. Future research should focus on developing custom neural networks trained on reliable 2D image coordinates, and implementing temporal smoothing models (such as Kalman filters or recurrent neural networks) to suppress tracking outliers and prevent hand-topology inversion artifacts.

## 5. Conclusions

This study presented a biomechanical and metrological validation of an integrated monocular markerless kinematic tracking pipeline (*N* = 10) against an 8-camera optoelectronic reference system across three camera viewpoints and four upper-limb anatomical degrees of freedom. The findings demonstrate that measurement agreement and absolute accuracy in single-camera vision systems are governed by the geometric relationship between the camera sensor plane and the primary anatomical plane of motion.

Planar joint kinematics moving parallel to the image plane—most notably shoulder abduction—demonstrated moderate to good trajectory agreement (ICC = 0.63–0.82) and potential utility for gross clinical screening (RMSE = 9.00^∘^–11.23^∘^). Furthermore, positioning the camera at an oblique elevated perspective (Camera 2) maximized ROM agreement for transverse plane movements, such as shoulder rotation, compared to the frontal and oblique side perspectives. Finally, localized multi-segment rotations (forearm pronation) exposed architectural limits at the pose-hand boundary, where visual inspection revealed frequent topology inversions and severe error dispersion.

These findings outline the potential clinical utility of single-camera markerless systems. When camera placement is strategically optimized relative to the movement plane, monocular RGB tracking shows potential as an accessible instrument for gross clinical screening of planar range of motion in telerehabilitation contexts, pending further clinical validation. However, for high-precision multi-planar joint kinematics and fine distal forearm/hand rotations, current single-camera pipelines remain inadequate; future deployments must integrate 2D spatial shape regressions, temporal smoothing filters, or multi-view camera fusion to surpass the fundamental depth limits of monocular vision.

## Figures and Tables

**Table 1:** Mathematical Definitions of Upper-Limb Joint Angles.

Kinematic Task	Plane of Calculation	Anatomical Vector/Plane Reference	Algorithm Source
Shoulder Abduction	2D Frontal (XY)	*v_arm_*:*Shoulder* → *Elbow* *v_ref_*:*Vertical Axis* (*Y*)	Pose
Shoulder Rotation	2D Transverse (XZ)	*v_forearm_*:*Elbow* → *Wrist* *v_ref_*:*Forward Axis* (*Z*)	Pose
Elbow Flexion	3D Space	*v*_1_:*Shoulder* → *Elbow* *v*_2_:*Elbow* → *Wrist*	Pose
Forearm Pronation	3D Space	*v_arm_*:*Shoulder* → *Elbow* *n_hand_*:*Defined by Wrist*, 2*nd MCP*, 5*th MCP*	Hands & Pose

Note: MCP = Metacarpophalangeal Joint.

**Table 2:** Agreement and Error Analysis of Monocular MediaPipe Tracking Against the Optoelectronic Reference System across 12 Experimental Conditions (N = 10 participants).

Movement	Camera Viewpoint	*ICC*_2.1_ [95%CI]	RMSE [95%CI] (°)	Bias [95% CI] (°)	*p*-Value	95% LoA (°)
Shoulder Abduction	Camera 1	0.82 [0.33, 0.95]	11.12 [8.96, 13.28]	6.75 [0.89, 12.60]	0.029	[−9.34, 22.81]
	Camera 2	0.63 [0.07, 0.83]	9.00 [7.12, 10.88]	13.62 [6.67, 20.58]	0.002	[−5.42, 32.66]
	Camera 3	0.77 [0.12, 0.93]	11.23 [9.49, 12.98]	5.48 [−2.38, 13.35]	0.149	[−16.06, 27.02]
Shoulder Rotation	Camera 1	0.47 [0.17, 0.60]	22.52 [20.72, 24.33]	18.09 [9.88, 26.40]	0.001	[−4.56, 40.74]
	Camera 2	0.68 [0.04, 0.83]	17.68 [14.07, 21.29]	10.91 [1.68, 20.14]	0.025	[−14.41, 36.29]
	Camera 3	0.60 [0.18, 0.76]	18.06 [15.88, 20.24]	13.53 [5.87, 21.19]	0.003	[−7.51, 34.50]
Elbow Flexion	Camera 1	0.19 [0.01, 0.35]	23.75 [20.70, 26.81]	37.33 [34.48, 40.18]	<0.001	[30.11, 44.61]
	Camera 2	0.31 [0.01, 0.61]	24.65 [20.77, 28.52]	14.40 [5.18, 23.66]	0.007	[−9.12, 37.92]
	Camera 3	0.18 [0.01, 0.33]	30.92 [27.42, 34.41]	41.60 [38.78, 44.38]	<0.001	[34.52, 48.68]
Forearm Pronation	Camera 1	−0.08 [−0.56, 0.57]	30.11 [26.40, 33.83]	−5.58 [−20.13, 8.70]	0.384	[−45.47, 33.77]
	Camera 2	0.34 [−0.27, 0.70]	45.69 [40.34, 51.05]	1.38 [−12.21, 14.97]	0.862	[−36.32, 38.48]
	Camera 3	0.09 [−0.29, 0.39]	34.74 [31.90, 37.58]	19.99 [−6.59, 44.24]	0.132	[−54.91, 94.90]

Note: ICC = Intraclass Correlation Coefficient (ICC_2.1_, two-way random-effects model, single measures, absolute agreement) calculated on participant-level peak range of motion (N = 10). CI = Confidence Interval. RMSE = Root Mean Square Error computed across continuous time-series waveforms. Bias = Mean participant-level difference (MediaPipe − Vicon). LoA = 95% Limits of Agreement. *p*-value is two-tailed one-sample *t*-test against zero.

**Table 3:** Summary of engineering guidelines for monocular kinematic tracking based on movement geometry.

Geometry Class	Kinematic Task	Tracking Performance	Primary Source of Error	Engineering Solution
2D Frontal Planar	Shoulder Abduction	Moderate to Good Agreement	Perspective errors	Perpendicular camera alignment
2D Transverse Planar	Shoulder Rotation	Coarse Screening Only	Depth resolution limitations	Oblique elevated camera position
3D Multi-Planar	Elbow Flexion	Qualitative Tracking Only	Joint center definition mismatch	System offset calibration models
Multi-Segment Distal	Forearm Pronation	Unacceptable	Local model topology flips	2D shape regression frameworks

Note: Guidelines are established based on anterior and anterior-oblique camera perspectives (frontal, elevated oblique, and 45^∘^ anterior-oblique).

## Data Availability

The data presented in this study are available on request from the corresponding author due to institutional privacy restrictions.
